# Age- and size-related reference ranges: A case study of spirometry through childhood and adulthood

**DOI:** 10.1002/sim.3504

**Published:** 2009-02-28

**Authors:** T J Cole, S Stanojevic, J Stocks, A L Coates, J L Hankinson, A M Wade

**Affiliations:** 1 MRC Centre of Epidemiology for Child Health, UCL Institute of Child HealthLondon WC1N 1EH, U.K.; 2 Portex Respiratory Unit, UCL Institute of Child HealthLondon, U.K.; 3 Department of Respiratory Medicine, Hospital for Sick ChildrenToronto, Canada; 4 Hankinson ConsultingValdosta, Georgia, U.S.A.

**Keywords:** age-related reference ranges, GAMLSS, LMS method, skewness, spirometry, height, weight, allometry

## Abstract

Age-related reference ranges are useful for assessing growth in children. The LMS method is a popular technique for constructing growth charts that model the age-changing distribution of the measurement in terms of the median, coefficient of variation and skewness. Here the methodology is extended to references that depend on body size as well as age, by exploiting the flexibility of the generalised additive models for location, scale and shape (GAMLSS) technique. GAMLSS offers general linear predictors for each moment parameter and a choice of error distributions, which can handle kurtosis as well as skewness. A key question with such references is the nature of the age-size adjustment, additive or multiplicative, which is explored by comparing the identity link and log link for the median predictor.

There are several measurements whose reference ranges depend on both body size and age. As an example, models are developed here for the first four moments of the lung function variables forced expiratory volume in 1 s (FEV_1_), forced vital capacity (FVC) and FEV_1_/FVC in terms of height and age, in a data set of 3598 children and adults aged 4 to 80 years. The results show a strong multiplicative association between spirometry, height and age, with a large and nonlinear age effect across the age range. Variability also depends nonlinearly on age and to a lesser extent on height. FEV_1_ and FVC are close to normally distributed, while FEV_1_/FVC is appreciably skew to the left. GAMLSS is a powerful technique for the construction of such references, which should be useful in clinical medicine. Copyright © 2008 John Wiley & Sons, Ltd.

## 1. INTRODUCTION

Reference ranges play an important role in clinical medicine, with values that lie outside the reference range viewed as an indication for further investigation and/or treatment. Common examples of reference ranges arise in clinical chemistry [1], while age-related reference ranges of height or weight in children are presented as growth charts [2]. In adult medicine reference ranges are often assumed to be independent of age, while in paediatrics they usually depend on age reflecting the changing maturation of the individual over time.

The LMS method is a longstanding [3] and highly cited technique for constructing age-related reference ranges for skew data, whereby the first three moments of the measurement's frequency distribution are modelled as cubic smoothing spline curves in age [4]. The Box-Cox power λ to transform the data to near-normality, the median μ and the generalized coefficient of variation (CV) σ together define the moments as functions of age, hence the name LMS (λ — μ — σ). The method has been used to construct national growth references in many countries [5–7].

The more recent generalized additive modelling of location, scale and shape technique (GAMLSS) extends the LMS method in several ways [8]. It offers a choice of error distributions (rather than just one), it handles quite general linear predictors for each moment parameter (rather than limited to the single covariate age) and it is flexible in the choice of link between predictor and outcome (the LMS method uses the identity link for all three parameters).

The World Health Organisation (WHO), following a review of the methods available [9], chose to use GAMLSS for the analysis of its recently published growth standard [2]. Exploiting the flexibility of GAMLSS, the WHO statisticians were able to model the fourth moment of the distribution in addition to the first three, but found no evidence for non-normal kurtosis. As a result the final model involved just the first three moments, and so was effectively the LMS method.

In addition to depending on age, several outcome variables also depend on body size—three examples are spirometry [10], basal metabolic rate [11] and blood pressure [12]. Spirometry, a measure of lung function, increases with age in childhood (due to growth and maturation) and declines with age in adulthood (due to loss of elastic recoil), and it also depends on height as a proxy for chest size. Basal metabolic rate changes similarly with age, increasing in children and declining in adults, and in addition is related to body size through both weight and height. Blood pressure increases throughout life, and again is related to body size in the form of weight and height. Body size is hereafter called simply size.

Until now these outcome variables have been modelled using traditional regression methods, usually assuming homoscedasticity and normality of residuals, with the periods of childhood and adulthood analysed separately due to the problems of handling the child-adult transition. However, the flexibility of GAMLSS allows an extended class of models to be fitted, where the distribution of the outcome variable depends not only on age, including the child-adult transition, but also on one or more measures of size.

A key question in such analyses is the nature of the relationship between the outcome, size and age—is it additive or multiplicative? A common approach is to use multiple linear regression, which assumes that the size and age effects are additive [13]. However, Cole showed that modelling adult spirometry requires an age-height interaction [14], which can be expressed as a multiplicative relationship of the general form outcome ∝ size^*b*^ × *f*(age) [15]. Such a model implies a power or allometric law between outcome and size, as is widely used for body size scaling both between and within species [16]. The additive and multiplicative models can be fitted in GAMLSS using respectively, the identity link and the log link.

The aim here is to show how GAMLSS can be used to model age- and size-related reference ranges including departures from linearity, homoscedasticity and normality, while at the same time including appropriate adjustments for size. The method is illustrated using a data set of spirometry from childhood to old age [10]. Section 2 describes the data, Section 3 the development of the model, Section 4 the results and Section 5 is the discussion.

## 2. DATA

The data for the study are for ethnic white children and adults aged 4 to 80 years from four sources: U.S.A. (NHANES III) [17], Belgium [18], England [19] and Canada [20]. All the data were generated by qualified technologists and met the relevant national quality criteria. Table I summarizes the number of subjects by sex from each study, with the US data split into children and adults. The data were analysed separately for males and females, owing to their different patterns of size, growth and ageing.

**Table I:** Numbers of subjects by sex and study.

Centre	Country	Age	Males	Females
1	U.S.A. [17]	8–17	351	371
1	U.S.A. [17]	18–80	546	1005
2	Belgium [18]	5–19	166	150
3	England [19]	4–19	448	313
4	Canada [20]	4–18	110	138
	Total	4–80	1621	1977

The studies included several measures of spirometry, but just two are considered here: forced expiratory volume in 1 s (FEV_1_) and forced vital capacity (FVC). The two measures are obtained from a rapid expiration following a full inspiration, and FEV_1_ is the volume expired in 1 s while FVC is the total volume expired. The ratio FEV_1_/FVC is also clinically useful.

## 3. STATISTICAL METHODS

### 3.1. The LMS method and GAMLSS

The principle of the LMS method is that the distribution of the outcome variable *Y* is defined by three age-varying parameters λ, μ, σ such that the transformed outcome


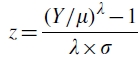
(1)

is a *z*-score with distribution close to N(0,1). This distribution, called by GAMLSS the Box-Cox-Cole-Green (BCCG) distribution, has properties such that the distribution is symmetric, i.e. any skewness in *Y* is removed by suitable choice of the Box-Cox power λ, the location parameter μ is hence the median rather than the mean and the scale parameter σ is approximately the dimensionless CV or log standard deviation (SD). Kurtosis is assumed to be absent.

GAMLSS is a generalization of the LMS method where *Y* has a specified frequency distribution *D*(μ, σ, ν, τ), the parameters representing the first four moments of the distribution. A wide variety of distributional forms are available, of which the normal distribution (called NO by GAMLSS) is the simplest with just two parameters, location (mean μ) and scale (SD σ). Other distributions have location, scale and one or two shape parameters (skewness ν and kurtosis τ), including the BCCG distribution (1) (where ν is equivalent to λ) and the Box-Cox-power-exponential (BCPE) distribution, which is an extension of the BCCG distribution to include kurtosis.

Each parameter of the distribution *D* is modelled as follows (this is for μ, the subscripts 1 corresponding to the first moment):


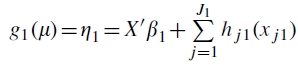
(2)

where *g*1(.) is the link function, *n*_1_ the linear predictor, *X'* β_1_ linear terms and 
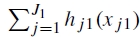
 additive terms (e.g. cubic splines) in the regression model. Corresponding equations define the other distribution parameters, i.e. σ, ν and τ for moments 2… 4. GAMLSS is best fitted using the package gaml ss [21] in R [22]. The age trends for each moment are fitted using cubic spline curves, as with the LMS method, which are more flexible than polynomials or fractional polynomials for modelling complex nonlinear relationships [23]. The complexity of the spline curve is defined in terms of equivalent (or effective) degrees of freedom (edf), and by R convention edf=0 corresponds to a linear term, while higher edf indicates a progressively more complex curve. The R model notation for a cubic spline of given edf is cs(*x*-variate, edf).

Model choice involves minimizing the Schwarz Bayesian Criterion SBC = deviance+df × log *n*, where the fitted deviance is —2 log Likelihood, *n* is the sample size and df is the total model degrees of freedom. The SBC is also used to choose the edf for individual spline curves. Thus, the SBC penalises the deviance by log *n* units for each extra df, leading to optimal spline curves and a parsimonious final model. The SBC is also known as the Bayesian information criterion. For comparison, the well-known Akaike information criterion (AIC) penalises the deviance by 2 units per extra df, which is a special case of the generalised AIC[k] or GAIC[k] where the penalty is *k* units of deviance per df. For data sets where log n>3 (as here), the AIC and GAIC [3] lead to less parsimonious models. The choice of SBC over AIC or GAIC [3] is justified in the present context by the visual appearance of optimal spline curves selected using the different criteria. GAIC-based curves are consistently and obviously under-fitted, whereas SBC-based curves are smoother and visually more convincing. This is acknowledged to be a subjective judgment, but it has the benefit of parsimony, which is important in such a rich modelling environment.

### 3.2. Modelling

Size is viewed here as one or more anthropometry variables such as height or weight. The same broad modelling issues apply to any reference range involving both size and age:

1. Are both size and age required in the model?
2. If yes, is the size-age relationship additive or multiplicative—should the size and age effects be added together or multiplied together?
3. What is the best way to model size—linear, log or power?
4. Is the age effect linear, quadratic or more complex?
5. Should variability be measured on the outcome scale (i.e. SD) or log outcome scale (CV)?
6. How does variability vary with age and size?
7. What is the distribution of the residuals, and does it vary with age and size?
8. Are there important technical effects, for example differences between the centres providing the data?

Taken together, these issues involve modelling the median (#1–4), the variability (#5–6) and the skewness and/or kurtosis (#7) of the distribution in terms of size and/or age. In addition technical differences may affect all four moments (#8). Note that since skewness is adjusted for, the location parameter is the median rather than the mean, though for the normal distribution of course the two coincide.

We now consider the moments of the model in turn, where size is represented by height.

### 3.3. Median μ

To predict the median, the questions above simplify to: (i) what are the best forms for modelling height and age (i.e. linear, log, (fractional) polynomial, cubic spline etc.)? (ii) are both the variables required? (iii) and if so, do they combine additively or multiplicatively?

The spirometry-height relationship is usually assumed linear in either height or log height, i.e. cs (Height, 0) or cs(log Height, 0), and slight nonlinearity can be modelled by increasing edf to 1 or 2. The relationship between height and log height is curved, so that cs(Height, 2) and cs(log Height, 2) are likely to fit very similarly.

Fitting age as a cubic spline allows for complex trends in childhood and adulthood, and it also models the transition between them. Polynomials or fractional polynomials do not provide sufficient flexibility. However, childhood is a much shorter period of time than adulthood (15 years as against 60 for the FEV_1_ data), so the spline curve struggles to capture the detail of the childhood phase while smoothing the adult phase. To make the two periods more equal, the splines are fitted on the transformed scale Age^*p*^ (or log age for *p* = 0), which for *p*<1 stretches the period of childhood and shrinks adulthood. The fitted curves are then redrawn on the original age scale. The optimal power transform is found by choosing *p* to minimize the deviance while keeping the age curve edf constant. If the deviance is reduced by less than log *n*, then *p* = 1.

Two separate models in height and age, additive and multiplicative, are fitted using the identity link


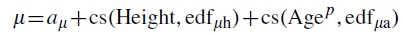
(3)

and log link


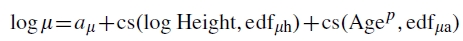
(4)

respectively, where edf_μh_ and edf_μa_ are the edf for height and age. Note that the log link uses log height to match conventional log-log regression, though height can be tested as an alternative. When edf_μh_ = 0 the models simplify to


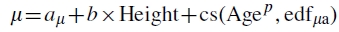
(5)

and


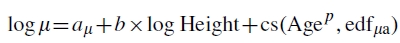
(6)

Coefficient *b* in (5) indicates the change in μ per unit change in height, while in (6) *b* shows the percentage change in μ per 1 per cent change in height. Antilogging (6) gives


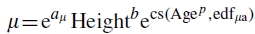
(7)

so that *b* is the height power, and μ is the product of height^*b*^ and an age term. This multiplicative model is in the form of the classic allometric growth curve.

As a further refinement *b* can be allowed to vary smoothly with age, where *b* is modelled as a cubic spline curve in (transformed) age with specified edf. This is known as a varying coefficient (vc in R) model [24]. Here *b* = vc(Age^*p*^, Height, edf_μah_) or *b* = vc(Age^*p*^, log Height, edf_μah_). For edf_μah_ = 0, the terms simplify to the linear interactions Age^*p*^ × Height or Age^*p*^ μ log Height, respectively.

Nearly all the FEV_1_-age-height models published to date have been special cases of (5) or (6), some of them including the age-height interaction. Age has been modelled as a low-order polynomial, often just a linear term, and the child and adult phases have been analysed separately. Thus, (3) to (6) represent a powerful family of models to apply to FEV_1_ throughout the life course from childhood to later life.

Other design covariates can be added to the model, for example a factor centre distinguishing the data sources to estimate the mean size of inter-centre differences after adjusting for height and age.

The nature of the fitted model and its goodness of fit can be assessed for each covariate in turn using term. plot(), which plots the fitted relationship and optionally includes the partial residuals (*zk—nk*)—see< ([8], Appendix B). The same can be done for the other moment parameters in the model.

### 3.4. Variability σ

Depending on the choice of distribution, the variability σ is measured either as the SD (in absolute units e.g. litres for FEV_1_) or the CV (in fractional units e.g. log FEV_1_), which correspond to the residuals from linear and log regression, respectively. GAMLSS can fit either—the normal distribution models the SD while the BCCG and BCPE distributions model the CV. In general, the CV is less variable than the SD across age, as in childhood the SD tends to vary in proportion to the median whereas the CV is relatively constant [4].

Previous spirometric models have assumed homoscedasticity of residuals, i.e. σ is independent of height and age. GAMLSS tests this by modelling σ in terms of height and age using the log link, analogous to (4)


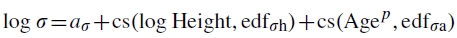
(8)

Note that for simplicity the same age transformation is used as for μ. Again design effects such as centre can also be added to model differential variability.

### 3.5. Skewness ν and kurtosis τ

Another common regression assumption is normality of the residuals, i.e. the residuals are assumed either normally or lognormally distributed, depending on the form of regression. With suitable choice of distribution, GAMLSS allows the degree of skewness and kurtosis to be modelled explicitly.

Non-normality is modelled using the BCPE distribution [8], where the skewness ν and kurtosis τ are estimated using models analogous to (3) and (4)


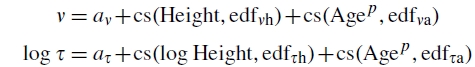
(9)

*Y* can be converted to a standardized random variable Z as in (1), where the values of μ, σ and λ (i.e. ν in (9)) are functions of height and age as described above. Z has a power exponential distribution with kurtosis parameter τ, and for a distribution with normal tails τ = 2.

A simple alternative to the BCPE distribution is the two-parameter normal distribution (NO), with mean μ and SD σ where *Y* is converted to a normally distributed *z*-score *z* = (*Y* — μ)/σ.

### 3.6. Three families

To explore the nature of the height-age relationship (additive versus multiplicative) and the residual error structure (CV versus SD), three different families of models are considered. Family 1 is an additive model based on the BCPE distribution with the identity link for μ (3). Family 2 is the multiplicative equivalent of family 1 with the log link for μ (4). Both families adjust for heteroscedasticity and non-normality by modelling CV σ (8) and ν and τ (9). Family 3 fits the normal distribution and models mean μ (3) and SD τ (8).

The analysis plan is to build the models incrementally, first estimating *p*, then μ assuming σ constant, ν=1 and τ = 2. Then σ is modelled to deal with heteroscedasticity, and then ν and τ are estimated to adjust for skewness and kurtosis (for families 1 and 2 only). Finally the effect of adjusting for centre is explored.

## 4. RESULTS

### 4.1. FEV_1_ in males

As a case study we describe the development of the regression model for FEV_1_ in males (*n* = 1621) in detail, and report the models for FVC and for females briefly.

#### 4.1.1. Median μ

The age parameters *p* and edf_μa_ain (3) and (4) are first optimised by direct search, across the three families, while setting edf_μh_ = 0 (i.e. including a linear height term). Table II summarizes the various models discussed here, one model per row, ranked in terms of decreasing SBC, where each extra parameter is penalised by log *n* ≈ 7.4 SBC units. The minimal SBC is 1325 units with family 2 (BCPE—log link), *p* = 0 (i.e. log age) and edf_μa_ = 7 (Table II row e)—the SBCs for *p* = ±0.1 and edf_μa_ = 6 or 8 are all greater. This age transformation reduces the SBC by 18 units (Table II rows d and e).

**Table II:** Development of the GAMLSS model for FEV_1_ in males (*n*=1621), with separate linear predictors for median μ, variability σ, skewness υ and kurtosis τ, where each row is a separate model.

		Linear predictor for μ						Linear predictor for log σ			Linear predictor for υ			
							
Model	Distribution	Link	p	edf_μa_ Height edf_μh_	Centre	edf_σa_	Height	Centre	υ	df	Deviance	SBC		
a	NO	identity	0	7	linear	0	–	–	–	–	1	12	1583.3	1672.0
b	BCPE	identity	0	7	linear	0	–	–	–	–	1	12	1292.4	1381.1
c	BCPE	log	0	7	linear	0	–	–	–	–	1	12	1262.0	1350.7
d	BCPE	log	1	7	log	0	–	–	–	–	1	11	1261.6	1342.9
e	BCPE	log	0	7	log	0	–	–	–	–	1	12	1236.3	1325.0
f	BCPE	log	0	7	log	0	–	2	log	–	1	16	1175.0	1293.3
g	BCPE	log	0	7	log	0	–	2	–	–	1	15	1177.0	1287.9
h	BCPE	log	0	7	log	0	–	2	–	–	constant	16	1163.5	1281.8
j	BCPE	log	0	7 log	0	yes	2	–	–	constant	19	1121.9	1262.3	
k	BCPE	log	0	7	log	0	yes	2	–	yes	constant	22	1096.7	1259.3
m	BCPE	log	0	7	log	0	yes	0	log	yes	constant	21	1097.3	1252.5

Note that τ=2 for all models. See text for details.

Families 1 (BCPE—identity link) and 3 (normal) fit much worse than family 2, with SBC 56 and 347 units greater, respectively (Table II rows b and a). For this reason the fitting focuses on family 2.

**Figure 1:**
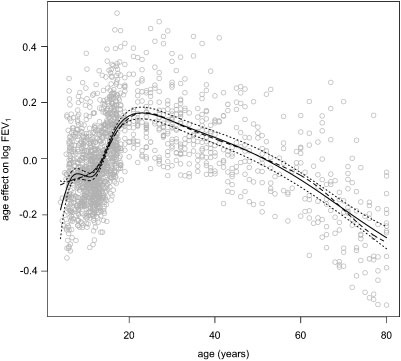
Age trend in the median (logμ) of FEV_1_ in males adjusted for height, with cubic spline curves fitted versus age (dashed line) and log age (solid line). The 95 per cent confidence interval for the solid curve (dotted lines) and the partial residuals about the solid curve are also shown. The curve shows a large and complex effect of age, rising in children and falling in adults.

Figure 1 compares the fitted spline curves for the models in rows d and e plotted on the age scale, with the partial residuals from the log age fit (row e). Figure 1 highlights not only the complexity of the child and adult trends and the child-adult transition, but also how transforming the age scale improves the fit in childhood. The y-axis, multiplied by 100, can be viewed as an approximate percentage scale [25]. The range of values in childhood exceeds 0.3, so that median FEV_1_ in boys aged 4 is 30 per cent less than in young men aged 23 after adjustment for height. Thus in childhood, age is very important over and above height.

**Figure 2:**
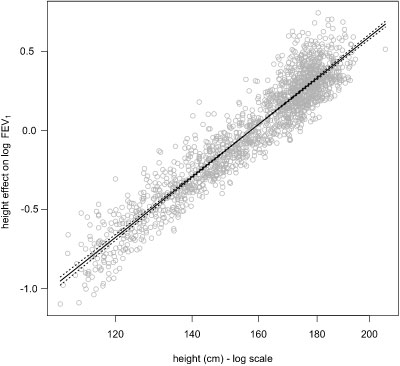
Log FEV_1_ versus log height in males after adjustment for age, with the linear regression fit (solid line) and its 95 per cent confidence interval (dotted lines), along with the partial residuals. After adjustment for age, the height effect is linear across the range.

Fitting height rather than log height in (4) increases the SBC by 26 units (Table II rows c and e), as does increasing edf_μh_. So edf_μh_ = 0 and log height is optimal. The log height regression coefficient *b* in (4) is 2.48 with standard error (SE) 0.034, so that adjusted for age, FEV_1_ goes as height^2.5^ at all ages. Figure 2 shows the partial regression plot for height with the fitted line.

Adding the log height by age interaction (edf_μah_ = 0) increases the SBC, and increasing to 1 increases it further, so there is no evidence for an interaction.

#### 4.1.2. Variability σ

**Figure 3:**
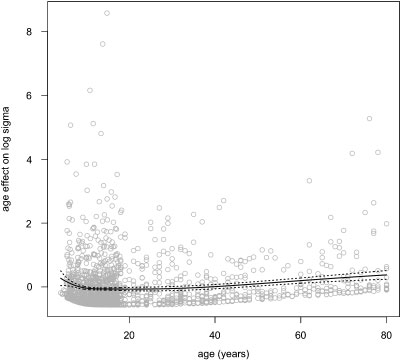
Age trend in the variability (logσ) of FEV_1_ in males, with the fitted cubic spline curve (solid line) and its 95 per cent confidence interval (dotted lines), along with the partial residuals. Variability is greater in young children and the elderly.

Modelling log σ (8) to adjust for heteroscedasticity, the optimal age curve has edf_σa_ = 2 with the log height term omitted, which reduces the SBC by 37 units (Table II row g). Figure 3 plots the partial residuals for log σ along with the fitted log age effect, and shows that the variability is greater below age 11 and in old age. Including log height in (8) increases the SBC (Table II row f).

#### 4.1.3. Skewness ν and kurtosis τ

**Figure 4:**
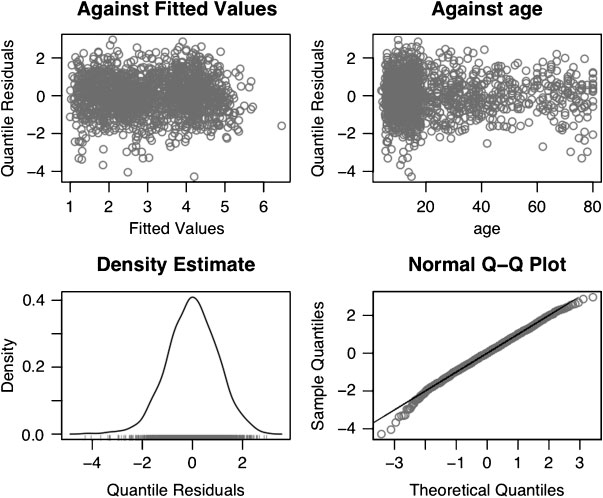
The residual distribution of FEV_1_ in males: quantile (standardised) residuals plotted against fitted values and against age, the density estimate with rug plot, and the quantile–quantile plot. The distribution is skew to the left.

Figure 4 shows the distribution of residuals for model g in Table II, where ν = 1 and τ = 2, and it indicates some left skewness. The two scatterplots show some low outliers, while the frequency distribution has a long left tail and the Q-Q plot shows negative skewness.

Optimizing v gives the value 1.59 SE 0.16, significantly greater than 1, which reduces the SBC by 6.1 units (Table II row h). It shows 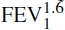 to be normally distributed after adjustment for height and age. Optimizing τ gives 1.85 SE 0.08, slight but insignificant non-normal kurtosis (i.e. τ ≠ 2). Neither height nor age (9) improves the prediction. Thus, the final model has 16 df (Table II row h) and the predictors are


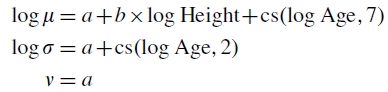
(10)

**Figure 5:**
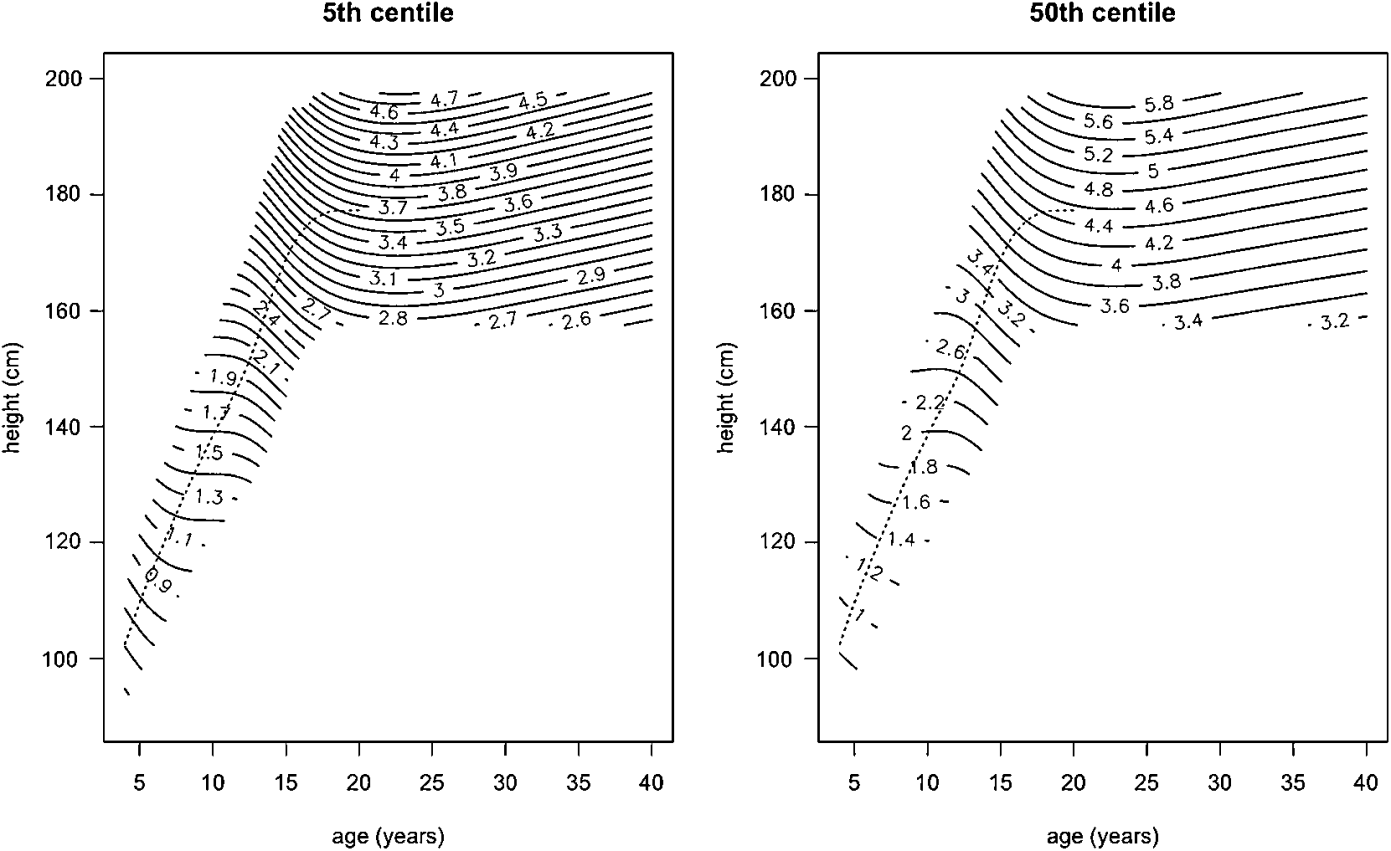
Contour plots of FEV_1_ versus age and height in males up to age 40. The dotted line is the median of the British 1990 height reference [26], and the plot is restricted to ±3 SDs about the median up to and beyond age 20. The two plots show the 5th and 50th centiles of predicted FEV_1_ as functions of age and height.

Figure 5 consists of two contour plots showing the combined effect of age and height on predicted FEV_1_ up to age 40 (the age range is truncated to emphasize the childhood phase). To restrict the prediction to plausible regions of the age-height plane, the range of heights by age is defined as ±3 SDs about the mean of the British 1990 height reference (shown as a dotted line in Figure 5) [26]. The plot shows the predicted 5th centile (the conventional lower limit of normal or LLN), and also the 50th centile.

It highlights the complex interplay between FEV_1_, age and height at different stages of childhood. The direction perpendicular to each contour shows the relative importance of height and age, so that between 8 and 12 years where the contours are essentially flat, height not age determines FEV_1_. At 140 cm for example, median FEV_1_ in this age range is 2L and the LLN is 1.6L, irrespective of age. At earlier and later ages the contours have negative slope in childhood, showing that both height and age are predictive. The pattern is simpler in adulthood where age and height are broadly uncorrelated and the parallel contours have positive slope.

**Figure 6:**
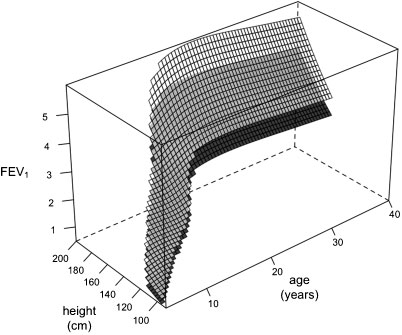
Wireframe plot of FEV_1_ in males up to age 40 predicted from age and height, the 5th and 50th centiles as in Figure 5.

Figure 6 shows the two contour plots of Figure 5 presented as surfaces in a 3-D wireframe plot. Unlike the contour plot, this provides qualitative rather than quantitative information, but it does give an idea of the surface shapes.

#### 4.1.4. Centre

The four centres are numbered as in Table I and adjusting for centre adds 3 df to the model for each moment predictor. Adjusting μ (4) reduces the SBC by nearly 20 units (Table II row j) and adjusting σ (8) reduces it by a further 2.9 (Table II row k). So there is an evidence of between-centre heterogeneity in both median and variability.

Adjusting σ for centre affects the predictor, so that the optimal choice of edf_σa_ = 2 with height omitted is challenged by a model consisting of age plus height, which reduces the SBC by 7 units to 1252.5 (Table II row m). The height effect is negative, which together with the positive age effect means that young children have increased variability due to their small size whereas older adults have increased variability due to their greater age.

Thus, if centre is included, there is an alternative final model with 21 df (Table II row m) and the three linear predictors are


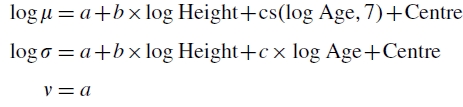
(11)

Table III gives the linear regression coefficients for (11). The log links for μ and σ mean that their coefficients are proportions and multiplied by 100 can be viewed as percentages. Thus for μ, the centre differences relative to centre 1 are −4 to +3 per cent, while for σ they are between −9 and +21 per cent. The log height coefficient for μ is close to 2.5, so that median FEV_1_ goes as height^2.5^. The estimate for ν of 1.5 is significantly greater than 1, indicating modest left skewness (as seen in Figure 5).

**Table III:** Linear regression coefficients for model (11) in row m of Table II.

	Estimate	SE	*t*	*P*
*μ link function: log*				
*μ coefficients:*				
Intercept	−11.4	0.15	−77.4	<0.0001
Log height	2.45	0.032	76.4	<0.0001
Centre 2	0.034	0.009	3.9	<0.0001
Centre 3	−0.038	0.007	−5.1	<0.0001
Centre 4	0.018	0.013	1.5	0.1
*σ link function: log*				
*σ coefficients:*				
Intercept	1.7	0.9	1.9	0.06
Log height	−0.95	0.20	−4.8	<0.0001
Log age	0.30	0.044	6.8	<0.0001
Centre 2	−0.090	0.064	−1.4	0.2
Centre 3	0.213	0.047	4.6	<0.0001
Centre 4	0.184	0.076	2.4	0.02
*υ link function: identity*				
*υ coefficients:*				
Intercept	1.50	0.16	3.1*	0.002*

* H_0_ :υ=1.

### 4.2. FVC and females

The optimal model for FVC in males is similar to that for FEV_1_, the only substantive difference being that FVC is normally distributed, with the estimate for ν close to 1. There is no evidence for centre differences.

FEV_1_ and FVC in females have a simpler age trend for μ than in males, but similar variability and a normal distribution. FEV_1_ also has a linear age-height interaction, with a steeper height slope in children than adults. Both FEV_1_ and FVC show centre differences for μ

**Figure 7:**
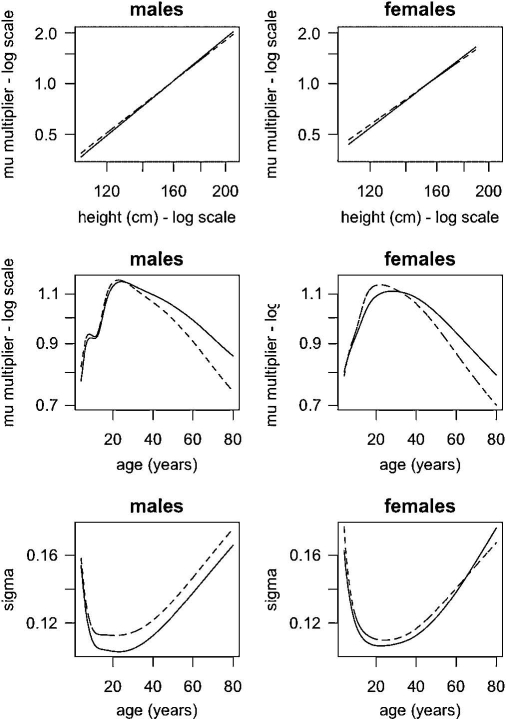
The effects of (a) height; (b) age on the median μ; and (c) age on the variability σ, of FEV_1_ (dashed lines) and FVC (solid lines) in males (left) and females (right).

Figure 7 shows the height and age effects on μ and σ for FEV_1_ and FVC in the two sexes, with the curves for FEV_1_ (dashed) and FVC (solid) superimposed. They confirm the similarity of the four height coefficients, all with *b*≈2.5, and the age trends are also similar for FEV_1_ and FVC within each sex.

### 4.3. FEV_1_ / FVC

Figure 7 shows that: (a) the height coefficient for FEV_1_ is slightly shallower than for FVC; (b) median FEV_1_ falls with age more steeply than median FVC; and (c) variability is slightly greater for FEV_1_ than for FVC. The linear predictor for FEV_1_/FVC is effectively the difference between the linear predictors for FEV_1_ and FVC, due to their log links, so that these discrepancies between them define the model for FEV_1_/FVC.

**Figure 8:**
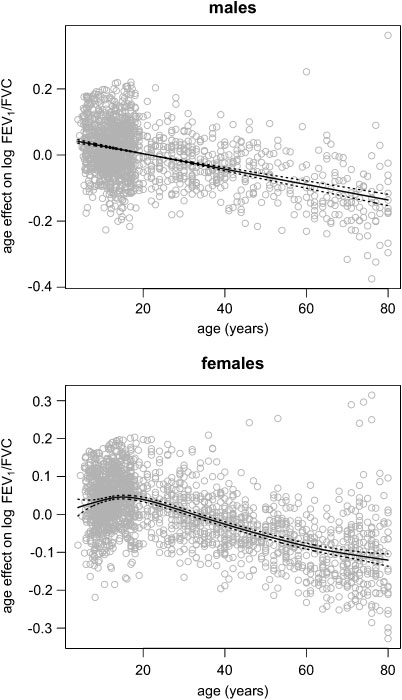
Age trends in the median (log μ) of FEV_1_/FVC by sex, adjusted for height, with fitted cubic spline curves (solid lines), 95 per cent confidence intervals (dotted lines) and partial residuals. The age trend is linear in males but non-linear in females.

In both the sexes, the log height coefficients for FEV_1_/FVC are tiny but highly significant, the age trends are broadly linear and highly significant and the variability is age-dependent. The distributions are very skew to the left with ν = 2.6 SE 0.24 in males and 2.4 SE 0.23 in females. Figure 8 shows the fitted age trends in FEV_1_/FVC by sex, adjusted for height, along with the partial residuals. The greater variability at the extremes of age is evident, as is the left skewness. Adjusting for centre differences reduces the SBC for both μ and σ

## 5. DISCUSSION

The study shows how GAMLSS can be used to construct reference ranges that depend on both age and size, and in the process adjust for departures from the four multiple linear regression assumptions of linearity, additivity, homoscedasticity and normality. Using spirometry as an example, the median μ of the distribution is found to vary nonlinearly with age, the relationship between outcome, size and age is multiplicative rather than additive, the CV σ varies nonlinearly with age and the distribution is left skew (though not kurtotic).

This is the first practical application of GAMLSS where the predictor is a function of more than one variable (setting aside the authors’ own worked examples [8,27]). The LMS method, which is a special case of GAMLSS where the predictor depends only on age, has been used for two decades to define the distribution of anthropometry (weight, height, etc.) by modelling age trends in the first three moments [3]. The present study extends the model to include size as well as age in the linear predictor, and also models the median using the log link, which allows the multiplicative model to be tested without forcing a lognormal error distribution. A multiplicative or power law model of the form outcome ∝ size_*b*_ × *f*(age) makes biological sense in terms of allometric scaling, and the results here confirm a far better fit for the log link than the identity link. **In detail the height coefficients *b* in (6) are all close to 2.5, so that the ratio indices FEV_1_ /height^2.5^** and FVC/height^2.5^ are broadly independent of height in both the sexes at all ages. A similar observation has previously been made for adults (with height^2^ rather than height^2.5^), but it clearly also applies to children [15].

GAMLSS was used here with the BCPE distribution, where the fourth parameter τ models kurtosis. The simpler three-parameter alternative, the BCCG distribution, corresponds to that of the LMS method and assumes no kurtosis. The fact that no kurtosis was detected supports the WHO conclusion that it need not be modelled [2] and that an adjustment for skewness is sufficient.

Evidence of left skewness was strongest for FEV_1_/FVC, where the Box-Cox power ν was around 2.5 in both sexes. As a ratio FEV_1_/FVC has an upper limit of 100 per cent, which inevitably means that the upper tail of the distribution is shorter than the lower tail and the adjustment for skewness compensates for this in a natural way. The skewness seen in FEV_1_ for males was absent for females and for FVC, and it was in any case only weak. We recommend that in practice skewness not to be adjusted for with FEV_1_ or FVC.

The existence of significant centre differences is important for generalizability and it emphasizes the need to standardize as far as possible the protocols used for spirometry measurement, in order to minimize centre differences. This is of course a requirement for any data collated from several sources, not just spirometry.

A real concern with GAMLSS is that its power and flexibility can lead to the development of complex models that lack biological plausibility—indeed the GAMLSS manual has a warning to this effect on page 2. Furthermore, 20 years ago the first author, introducing the LMS method, wrote

Producing centile charts has always been something of a black art—the centile lines need to be drawn such that they are both smooth and close to the empirical centiles. It is not surprising that this trade-off problem is often solved by drawing the lines by eye.

The march of progress means that although the same sentiment still applies, the uncertainty now lies in the choice of model rather than the absence of one. The use of the SBC rather than the AIC or GAIC [3] for model choice results in more parsimonious final models, which provides some protection against this. Also the distributions considered here (BCPE, BCCG and normal) are just three of the 40-plus available in gamlss [27], but they are tried and tested. Also we justify our form of body size adjustment in biological terms. Taken together these constraints act as prior information to reduce the number of models to be considered. It is still possible that our model will be sub-optimal in certain situations, but we believe that the (probably only slightly) poorer fit will be offset by the robust pre-specified modelling framework.

The value of a parsimonious model adjusting for size and age is that the outcome measure can be expressed as an adjusted *z*-score or centile (1), which is valuable for interpretation by clinicians. We have published a clinical paper describing our lung function model [10], and an independent review has described the major impact it will have on clinical practice [28]. The downside of a combined size-age adjustment is that the reference ranges cannot, as is routinely done with growth centile charts, be presented graphically on a 2-D chart. The extra dimension requires a 3-D chart that ideally needs to be computerised. Figures 5 and 6 give an idea of what can be displayed in 2-D, but they are inadequate for detailed work. We have developed a Microsoft Excel add-in module called LMSGrowth that does the measurement to *z*-score conversion, either for individuals or groups [29].

In conclusion the study has demonstrated how age-related reference ranges can be extended to include an adjustment for size, by modelling the distribution in terms of its first three moments, using the log link for the median.
